# Helmet wearing behavior where people often ride motorcycle in Ethiopia: A cross-sectional study

**DOI:** 10.1371/journal.pone.0262683

**Published:** 2022-01-27

**Authors:** Delwana Bedru, Firanbon Teshome, Yohannes Kebede, Zewdie Birhanu

**Affiliations:** 1 Department of Public Health, College of Health Science and Medicine, Werabe University, Werabe, Ethiopia; 2 Department of Health, Behavior and Society, Faculty of Public Health, Jimma University, Jimma, Ethiopia; Tsinghua University, CHINA

## Abstract

**Background:**

Road traffic accidents are a major global concern that affects all people regardless of their age, sex, wealth, and ethnicity. Injuries and deaths due to motorcycles are increasing, especially in developing countries. Wearing helmet is effective in reducing deaths and injuries caused by motorcycle accidents.

**Objectives:**

To assess the magnitude of helmet wearing behavior and its determinants among motorcycle riders in Sawula and Bulky towns, Gofa zone, Southern Ethiopia.

**Methods:**

A community-based cross-sectional study was conducted from April, 15 to May 25, 2020, among 422 motorcycle drivers in Sawula and Bulky towns, where people often drive motorcycles. A stratified sampling technique was used to recruit sampled drivers in a face-to-face interview. Data were entered into EPI-data version 3.1 software and exported to SPSS version 23 software to manage analysis. Descriptive analyses such as frequency, percentage, mean and standard deviation were performed as necessary. Logistic regression models were fitted to identify the predictors of helmet wearing behavior. Adjusted odds ratios (AOR) with 95% confidence interval (CI) were used to determine the magnitude and strength of the association.

**Results:**

A total of 403 motorcycle drivers participated in the study which gave a 95.5% response rate. Among 403 motorcycle riders, only 12.4% (95% CI, 9.2 to 15.6%) wore helmets while driving motorcycles. Having license [AOR 3.51(95% C.I 1.56–7.89)], driving distance >10Km [AOR 2.53(95% C.I 1.08–5.91)], History of exposure to accident [AOR 2.71(95% C.I 1.32–5.55)], driving experience of ≥10 years [AOR 2.98 (95% C.I 1.25–7.09)] and high perceived susceptibility to accident [AOR 3.10(95% C.I 1.29–7.46)] had statistically significant association with helmet wearing compared to their counterparts.

**Conclusions:**

This study found that helmet-wearing behavior was very low. Having a license, driving distance, exposure to accidents, driving experience, and accident risk perception were determinants of helmet wearing behavior. These determinants imply the need for interventions that focus on behavioral change communications such as awareness creation campaigns and mandatory helmet wearing laws.

## Background

Road traffic accidents (RTAs) are among the leading causes of deaths, disabilities, and injuries worldwide [1, 2]. Every year, as many as 50 million people are injured and nearly 1.3 million people lose their lives on the road [3]. Road injuries are the 8^th^ leading cause of death globally, in developing countries and in sub-Saharan Africa. In contrast, they are the 17^th^ leading cause of death in developed countries even though they have dramatically higher motorization rates [4, 5]. Despite the low number of registered vehicles, the burden of RTA is disproportionally high in low- and middle-income countries, where over 90% of the world’s fatalities on roads occur in these regions [6, 7]. The road injury death rate in sub-Saharan Africa (27.0 per 100,000 people) was 40% higher than the global rate. Four countries (Nigeria, Ethiopia, South Africa, and Sudan) together account for half the road injury death toll of sub-Saharan Africa [4]. In Ethiopia, the rates of injuries and fatalities secondary to RTAs are exceptionally high and more than one-third of such accidents occur in vulnerable road users including motorcyclists, pedestrians, and cyclists [8].

Globally, motorcycles account for 23% of RTA-related deaths [2]. Motorcycle crashes are among the leading causes of traffic-related injuries and deaths in all age groups, especially in low- and middle-income countries [9–12]. This can be attributed to the use of motorcycles for commercial transport [2]. Motorcycles are the most popular private transportation vehicles in low-income countries. Evidence has shown that motorcycle drivers are among the most vulnerable road users [1, 13]. In India, the prevalence of motorcycle injuries was 56.1% [13]. In Sub-Saharan Africa, motorcycle driver death accounts for 13% of RTA-related deaths [14]. The problem is much higher in some countries. For instance, it constitutes 37.2% of all road traffic injuries in Tanzania [15], about 33% of fatalities associated with RTA in Ugandan, 24% in Kenya, and 18% in Ghana [1]. In Ethiopia, 21.0% of the deaths from RTA causalities were motorcycle drivers [8]. Evidence from studies conducted in Ethiopia indicated that the death rate due to a motorcycle crash in some areas was higher than that in the national figure. For instance, cross-sectional studies from Arbaminch and Wolayita showed that 40% and 31.2% of road traffic injuries resulted from motorcycle crashes, respectively [16, 17].

In addition to death and disabilities; motorcycle accident-related injuries can also result in economic losses for individuals, families, and communities due to the requirement of specialized or long-term care, medical costs, loss of labor outputs, and funeral expenses [18–20]. Motorcycle crashes have a greater risk of serious injuries and death than to other forms of transport because of the body structure of the vehicle and motorcycle users lack of protection equipments such as seat belts, helmets and airbags in the event of crashes [21, 22].

Evidence indicates that wearing a helmet is the single most effective way of reducing serious head and brain injuries, and its negative consequences including fatalities resulting from motorcycle crashes [1, 23–25]. For instance, a systematic review showed that helmets are effective in reducing the risk of head injuries in motorcyclists by 69% and death by 42%, resulting in a significant reduction in the healthcare costs associated with a crash [26]. A study from Addis Ababa Ethiopia also showed that the severity of injury was high among motorcycle riders or passengers without helmets [27]. In addition, wearing helmets reduces the length of hospital stay and medical costs of injured riders [28–30]. Indeed, it also helps to protect teeth and face from injury, increase visibility due to its reflectivity, help protect eyes from the effects of direct sun’s rays or rain and blocks the cool breeze from entering ears [30, 31]. Thus, helmet-wearing highly contributes to the achievement of the sustainable development goal-3.

Despite, the effectiveness of helmet wearing in reducing death, disabilities, injuries, psychological, economic and societal impacts due to motorcycle accidents; to the best of the authors’ knowledge, the status of helmet wearing behavior was unknown in Ethiopia. Therefore, this study aimed to assess helmet wearing behavior and its determinants among motorcycle drivers in Sawula and Bulky towns, Gofa zone, Southern Ethiopia, 2020.

## Methods

### Study design, period and setting

A community-based cross-sectional study was conducted from April 15 to May 25, 2020 in Sawula and Bulky towns. Sawula and Bulky towns are found 514 Km and 531 Km away from Addis Ababa (the capital city of Ethiopia), respectively. Based on the 2020 report obtained from the Sawula and Bulky towns’ health offices, Sawula town has ten kebeles (the smallest administrative unit next to district) and Bulky town has five kebeles. Sawula and Bulky towns had a total population of 46,957 and 25,000; and households 9,582 and 5,201 respectively [32, 33]. Sawula town has one governmental general hospital, one health center and twelve private clinics [32]. Bulky town has one health center and four private clinics [33]. Motorcycles are the common mode of transportation in both Sawula and Bulky towns. The two towns were purposely selected based on researchers’ familiarity with the study area and resource issues. Both towns are rapidly increasing in population size and economic growth among the towns of the Gofa Zone, Southern Ethiopia.

### Population

The source populations were all motorcycle drivers in the Sawula and Bulky towns. The study population consisted of randomly selected motorcycle drivers who were driving motorcycles in the three months prior to the study period. Motorcycle drivers who were driving motorcycles in the three months prior to the study period were included in the study. In contrast, motorcycle drivers who were unable to communicate or severely ill during the data collection period, those who lived in the study areas for less than six months and those who left the towns for different reasons during the data collection period were excluded.

### Sample size determination and sampling procedure

The sample size was calculated using a single population proportion formula with the assumption of 50% proportion of helmet wearing behavior (since there were no studies in Ethiopia that can address this objective), 1.96 standard normal distribution curve value for 95% level of confidence, and 5% margin of error between the sample and the population. Finally, considering a non-response rate of 10%, the total sample size was calculated to be 422.

The participants of the study were selected as follows: First, the sampling frame was constructed after obtaining the lists of owners of motorcycles, their phone number, and motorcycles’ plate numbers from the registration books of Sawula and Bulky towns’ road and transportation office. Accordingly, 500 and 250 motorcycle owners were identified in Sawula and Bulky town, respectively. Then, the sample size was allocated proportionally to the two towns. Finally, a computer-generated simple random sampling technique was used to identify the study participants. Phone numbers were used to contact the participants. For those whose phone calls were not working, their usual place of residence and working area was identified in collaboration with health extension workers. Since the possibility that a motorcycle might not be ridden by the owner and/or the existence of more than one rider for a single motorcycle; individuals who commonly drive the motorcycle during the last three months before data collection were selected after obtaining information from the owners of motorcycles. In case when difficult to know who commonly drive the motorcycle, a lottery method was used to select them.

### Data collection tools and procedures

An interviewer-administered structured questionnaire was adapted from the literatures [34–37]. It was initially prepared in English and then translated into local languages (Gophigna and Amharic) and back-translated into English by an independent translator to check for consistency of meaning. The questionnaire comprised seven parts: socio-demographic and economic characteristics, driving-related factors, substance use, knowledge about helmets, perceptions (perceived susceptibility and perceived severity), social pressures, and questions related to helmet wearing behavior. Perceived susceptibility, perceived severity, and social pressures were assessed using a five-point scale response format, where: 1 = strongly disagree, 2 = disagree, 3 = neutral, 4 = agree, 5 = strongly agree). Perceived susceptibility and perceived severity were assessed using four items, and social pressure related to helmet wearing was assessed using five items. Knowledge about helmets was assessed by three items in the ‘Yes or No’ format. A correct answer was coded as “1” and an incorrect answer was coded as “0”. Helmet wearing behavior was assessed by two questions: One yes or No question (Have you used helmet prepared for motorcycle drivers in the past three months during driving?) and for those who answered “yes” to the first question, they were asked an additional three-point Likert scale question (How often do you wear?) with response options of 1 = Rarely, 2 = Sometimes and 3 = Always. A pretest was conducted on 5% of the total sample size in the shefite town and some modifications were made based on the findings. The internal consistency of the items was checked using Cronbach’s alpha. Accordingly, the alpha of knowledge α = 0.81, perceived susceptibility α = 0.98, perceived severity α = 0.85 and social pressures α = 0.91. In this study, the face and content validity of the instrument was mainly focused on obtaining suggestions from experts and primary target audiences/motorcycle drivers. The questionnaire was presented to three public health experts. Their suggestions were considered during the modification of the questionnaire. In addition, during the pre-testing of the draft instrument, participants were invited to comment and give suggestions on the clarity, simplicity, language, phrasing, and how well the questionnaire matched their experiences. In this study, other types of validity were not addressed.

Six data collectors (four BSc Nurses and two diploma Nurses) and two supervisors (Health Officers) were involved in the study. One day intensive training was given to data collectors and supervisors on the aim of the study, data collection tools, research ethics, and approaches to study participants. Data collection was conducted at workplaces, main parking areas, and areas where the traffic police monitor the drivers/police stations. Close supervision during data collection, daily feedback, and proper cleaning before and after entry were performed. In addition to supervisors, the authors coordinated the data collection, made site visits, and oversaw the whole process and then if any inconsistency and errors were checked and solved immediately.

### Study variables

Helmet wearing behavior was the dependent variable. Socio-demographic and economic factors (age, educational level, marital status, main occupation, and monthly income), driving-related factors (driving license, driving experience, motorcycle accident, driving distance and frequency), substance use-related factors (alcohol drinking and khat chewing), perceptions (perceived susceptibility and perceived severity), knowledge and social pressures related to helmet wearing were independent variables.

### Operational definition and measurements

In this study, motorcycle riders were considered “wearing helmets” if they were always wearing helmets for the sake of reducing motorcycle injuries while driving in the last three months before the study period. This means, respondents who *“hadn’t worn*, *wear rarely and wear sometimes*” were considered as “Not wearing helmet” and those who *“wear always”* were considered as “Wearing helmet”.

To measure perceived susceptibility, perceived severity, and social pressure; subscale scores were computed by summing item scores and dividing by the total number of items. Then, dichotomization was made by taking the mean as a cut-off point. Scores above or equal to the mean were considered as “high” and scores below the mean were considered as “low”. Knowledge about helmets was measured by computing the total score after summing all three items together. Then, scores above or equal to the mean value were considered as “good knowledge” scores below the mean value were considered as “poor knowledge”.

#### Social pressure

Any influence on motorcycle drivers from his friends and/or families and/or community to wear helmets.

### Data processing and analysis

The collected data were entered into Epidata version 3.1 statistical software and then sorted, coded, checked, cleaned, and analyzed using SPSS version 23 software. Descriptive statistics such as frequencies, proportions, means and standard deviations were used to summarize the findings. Binary logistic regression analysis was performed to select variables for the multivariable regression analysis. Accordingly, variables with a p-value < 0.25 in the binary logistic regression analysis were considered as candidates for multivariable regression analysis. Finally, multivariable logistic regression analyses were performed to control for possible confounding effects of the selected variables. Crude and adjusted odds ratios, and 95% confidence intervals (CIs) were used to determine the magnitude of the association. Variables with a p-value of < 0.05 were considered as statistically significant determinants for wearing helmets. Model fitness was checked using the Hosmer and Lemeshow goodness-of-fit test and the model test P-value was found to be 0.10. Finally, the results were presented in the form of tables, figures and narratives.

### Data quality assurance

The questionnaire was prepared in English and then translated into local languages and translated back into English by another person to check its semantic equivalence. The questionnaire was pre-tested on 5% of the sample size. Training was provided to the data collectors and supervisors. In addition, the data collection process was closely supervised by the authors to evaluate the completeness and consistency of the data, and provide daily feedback. The reliability of the tool was also evaluated. The face and content validity of the tool were approved by experts and primary target audiences.

### Ethical approval and consent to participate

Ethical clearance was obtained from the Research and Ethical Review Committee of Jimma University. A permission letter was secured from Sawula and Bulky town Health Offices, and road and transportation offices. Written informed consent was obtained from each participant. Written consent was obtained from the parents of the participants under 18 years of age. All participants were informed of the purpose and benefits of the study. They were informed that participation in the study was voluntary and that they could refuse to participate or withdraw from the study without any penalties. Moreover, the participants were reassured that their responses would remain confidential.

### Schematic presentation of methods

**Fig 1:**
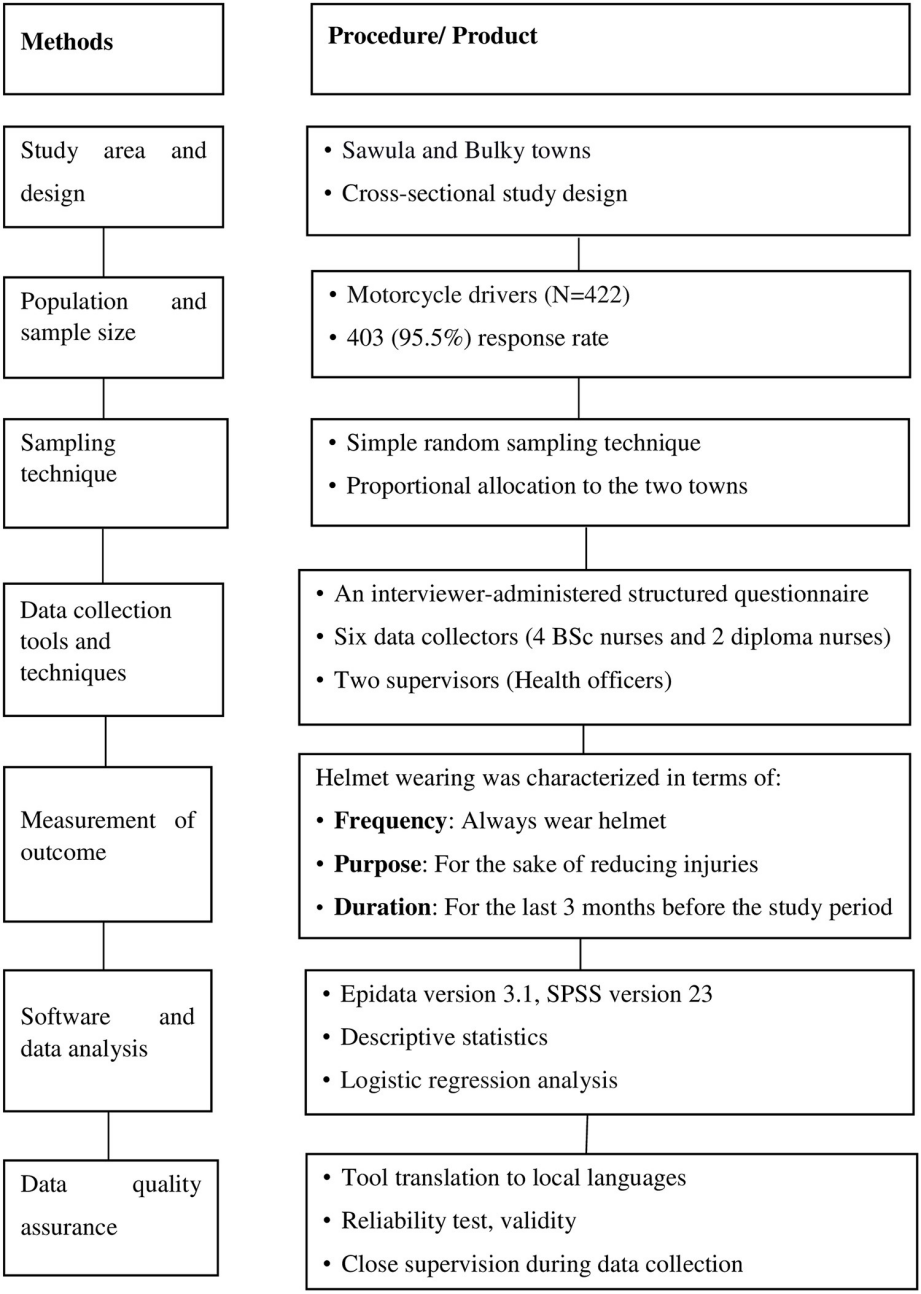
Methods structure diagram.

A diagram was used to explain the main content of the methods section (Fig 1).

## Results

### Socio-demographic and economic characteristics of the respondents

A total of 403 motorcycle drivers participated in the study which giving a response rate of 95.5%. A majority, 246(61.0%) of the respondents were in the age range of 18–35 years. More than half, 226(56.1%) were single by marital status. Of participants, 203(50.4%) were commercial motorcycle drivers. Of the 403 respondents, 373 (92.6%) attended formal education (Table 1).

**Table 1:** Socio-demographic and economic characteristics of motorcycle drivers in Sawula and Bulky towns, Gofa zone, Southern Ethiopia, 2020.

Variable	Category	Frequency	Percentage
Age (yrs.)	<18	54	13.4
	18–34	246	61.0
	≥35	103	25.6
Marital status	Single	226	56.1
	Married	148	36.7
	Divorced	17	4.2
	Widowed	12	3.0
Educational status	No formal education	30	7.4
	Primary (1–8)	133	33.0
	Secondary (9–12)	81	20.1
	Technical/vocational	48	11.9
	Degree and above	111	27.5
The main occupation of the respondents	Commercial motorcycle driver	203	50.4
	Farmer	44	10.9
	Merchant	66	16.4
	Government employee	73	18.1
	Non-governmental employee	17	4.2
Monthly income	<1000	22	5.5
	1000–2000	66	16.4
	2000–3000	96	23.8
	3000–4000	80	19.9
	>4000	139	34.5

### Driving and substance use-related factors

Of a total of 403 motorcycle drivers, 202(50.1%) had motorcycle driving licenses. The majority of them, 251(62.3%) and 280(69.5%) had a history of driving experience of less than five years and drive commonly more than ten kilometers, respectively. More than one-fourth, 110(27.3%) of the respondents had a history of exposure to a motorcycle accident. More than three-fourths, 306(75.9%) of the participants had a history of alcohol drinking (Table 2).

**Table 2:** Driving and substance use-related factors among motorcycle drivers in Sawula and Bulky towns, Gofa zone, Southern Ethiopia, 2020.

Variable	Category	Frequency	Percentage
Had license	Yes	202	50.1
	No	201	49.9
Driving experience (yrs.)	<5	251	62.3
	5–9	99	24.6
	≥10	53	13.2
Driving distance	≤10Km	123	30.5
	>10 Km	280	69.5
Driving frequency	Daily	181	44.9
	Sometimes	149	37.0
	Rarely	73	18.1
Ever had exposure to a motorcycle accident	Yes	110	27.3
	No	293	72.7
History of alcohol drinking	Yes	306	75.9
	No	97	24.1
History of khat chewing	Yes	297	73.7
	No	106	26.3

### Perceptions, knowledge and social pressures

The mean scores for perceived susceptibility, perceived severity, knowledge about helmets and social pressures related to helmet wearing were summarized in Table 3.

**Table 3:** Perceptions, knowledge and social pressures related to motorcycle accident and helmet wearing in Sawula and Bulky towns, Gofa zone, Southern Ethiopia, 2020.

Variable	Minimum	Maximum	Range	Mean [Std. deviation]
Perceived susceptibility	7	20	13	15.59[±2.21]
Perceived severity	4	20	16	14.96[±2.20]
Knowledge	1	3	2	2.10 [± 0.33]
Social pressure	8	25	17	15.77[±4.13]

### Prevalence of helmet wearing behavior

**Fig 2:**
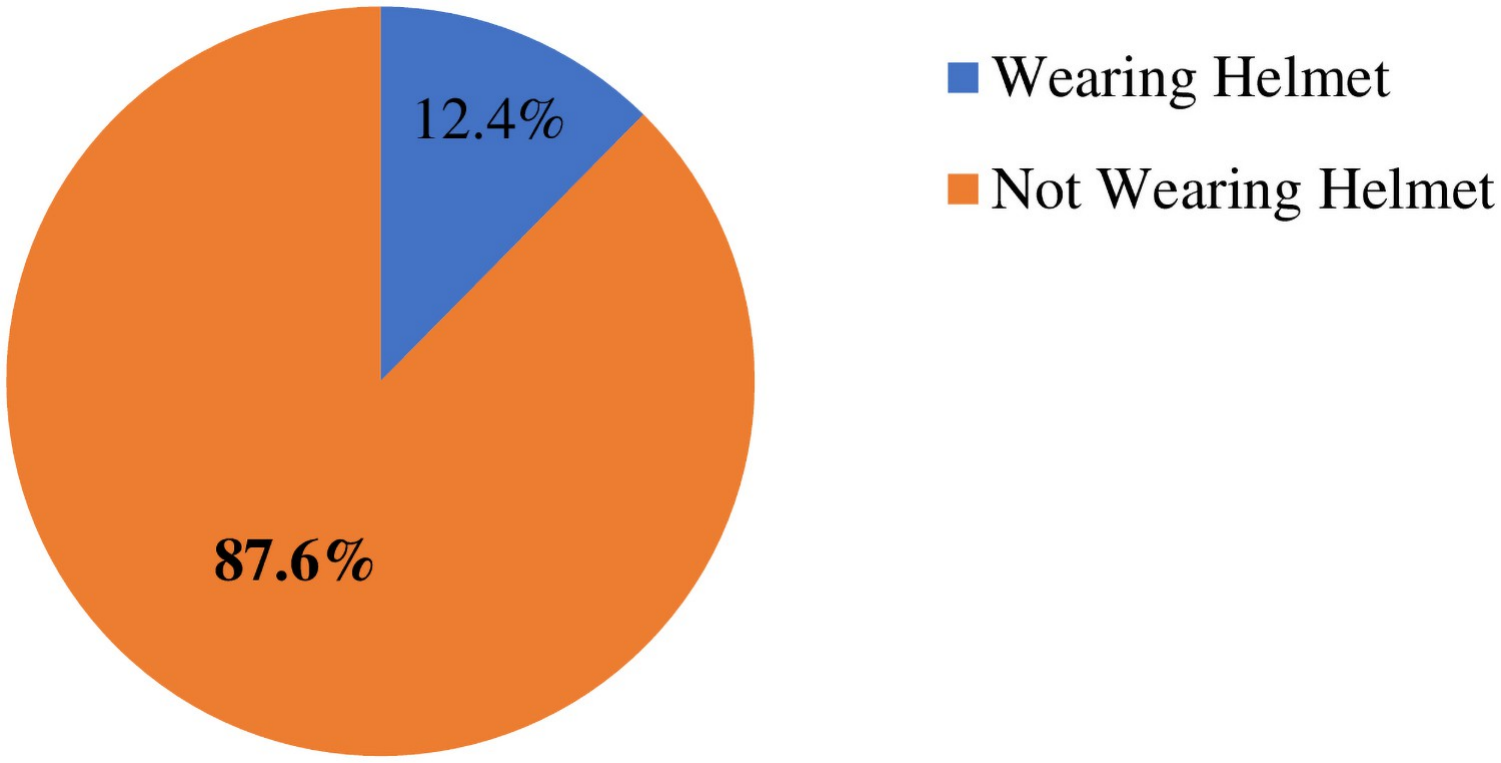
Prevalence of helmet wearing among motorcycle riders in Sawula and Bulky towns, Gofa zone, Southern Ethiopia.

The findings of this study indicated that among a total of 403 motorcycle riders, only 50(12.4%) wore helmets while driving a motorcycle (Fig 2).

### Factors associated with motorcycle drivers’ helmet wearing behavior

All independent variables were checked for their association with helmet wearing in binary logistic regression. As shown in Table 4, a total of eleven variables (age, having license, driving distance, driving area, history of motorcycle accident, history of alcohol drinking, driving experience, knowledge about wearing helmet, social pressure, perceived susceptibility and perceived severity) had a significant association with helmet wearing at p-value <0.25 in binary logistic regression analysis. However, after adjusting for potential confounders in multiple variable logistic regression analysis, having license, driving distance, history of exposure to motorcycle accidents, driving experience and perceived susceptibility were found to be statistically significant predictors of helmet wearing in motorcycle drivers.

**Table 4:** Logistic regression analyses of factor associated with helmet wearing among motorcycles drivers in Sawula and Bulky towns, Gofa zone, Southern Ethiopia, 2020.

Variable	Helmet wearing status		COR (95%CI)	AOR (95% CI)
	Wear	Not wear		
**Age (yrs.)**				
<18	1	53	1	1
18–34	28	218	6.81[.91–51.17]	2.97[0.37–24.17]
≥35	21	82	13.57[1.77–103.93] *	4.56[0.52–39.69]
**Have license**				
Yes	41	161	5.43[2.56–11.52] **	3.51[1.56–7.89] *
No	9	192	1	1
**Driving distance**				
≤10Km	8	115	1	1
>10Km	42	238	2.54[1.15–5.58] *	2.53[1.08–5.91] *
**Common driving area**				
Outside the main road	15	164	1	1
On the main road	35	189	2.03[1.07–3.84] *	1.55[0.76–3.16]
**History of accident**				
Yes	26	84	3.47[1.89–6.36] **	2.71[1.32–5.55] *
No	24	269	1	1
**History of alcohol drinking**				
Yes	42	264	1	1
No	8	89	0.57[0.26–1.25]	0.45[0.19–1.08]
**Driving experience**				
<5 yrs.	20	231	1	1
5–9 yrs.	17	82	2.40[1.20–4.79] *	1.78[0.83–3.83]
≥10 yrs.	13	40	3.75[1.73–8.15] *	2.98[1.25–7.09] *
**Knowledge**				
Poor	17	160	1	1
Good	33	193	1.61[.86–3.00]	1.29[0.63–2.67]
**Social pressure**				
Low	13	146	1	1
High	37	207	2.01[1.03–3.91] *	1.32[0.61–2.85]
**Perceived susceptibility**				
Low	7	130	1	1
High	43	223	3.58[1.57–8.19] *	3.10[1.29–7.46] *
**Perceived severity**				
Low	6	115	1	1
High	44	238	3.54[1.47–8.56] *	1.69[0.56–5.11]

*Statistically significant at P-value <0.05,

** statistically significant at P-value <0.001

Findings showed that the likelihood of wearing helmet were nearly 3.5 times [AOR 3.51(95% C.I 1.56–7.89)] higher among motorcycle drivers who had license compared to their counterparts. The driving distance also determined the helmet wearing. The odd of wearing helmet were nearly 2.5 times [AOR 2.53(95% C.I 1.08–5.91)] higher among those who drive a distance of greater than 10Km compared to those who drive distance of ≤10Km. Motorcycle drivers who had a history of motorcycle accidents were nearly 2.7 times [AOR 2.71(95% C.I 1.32–5.55)] more likely to wear helmets compared to those who had no history of motorcycle accidents. The findings of this study also showed the existence of an association between driving experience and helmet wearing. The odd of wearing helmet were nearly three times [AOR 2.98 (95% C.I 1.25–7.09)] higher among motorcycle drives who had a driving experience of ≥10 years compared to those who had a driving experience of <5 years. Motorcycle drivers who had high perceived susceptibility to motorcycle accidents were nearly three times [AOR 3.10(95% C.I 1.29–7.46)] more likely to wear helmets compared to their counterparts (Table 4).

### Connection between factors

**Fig 3:**
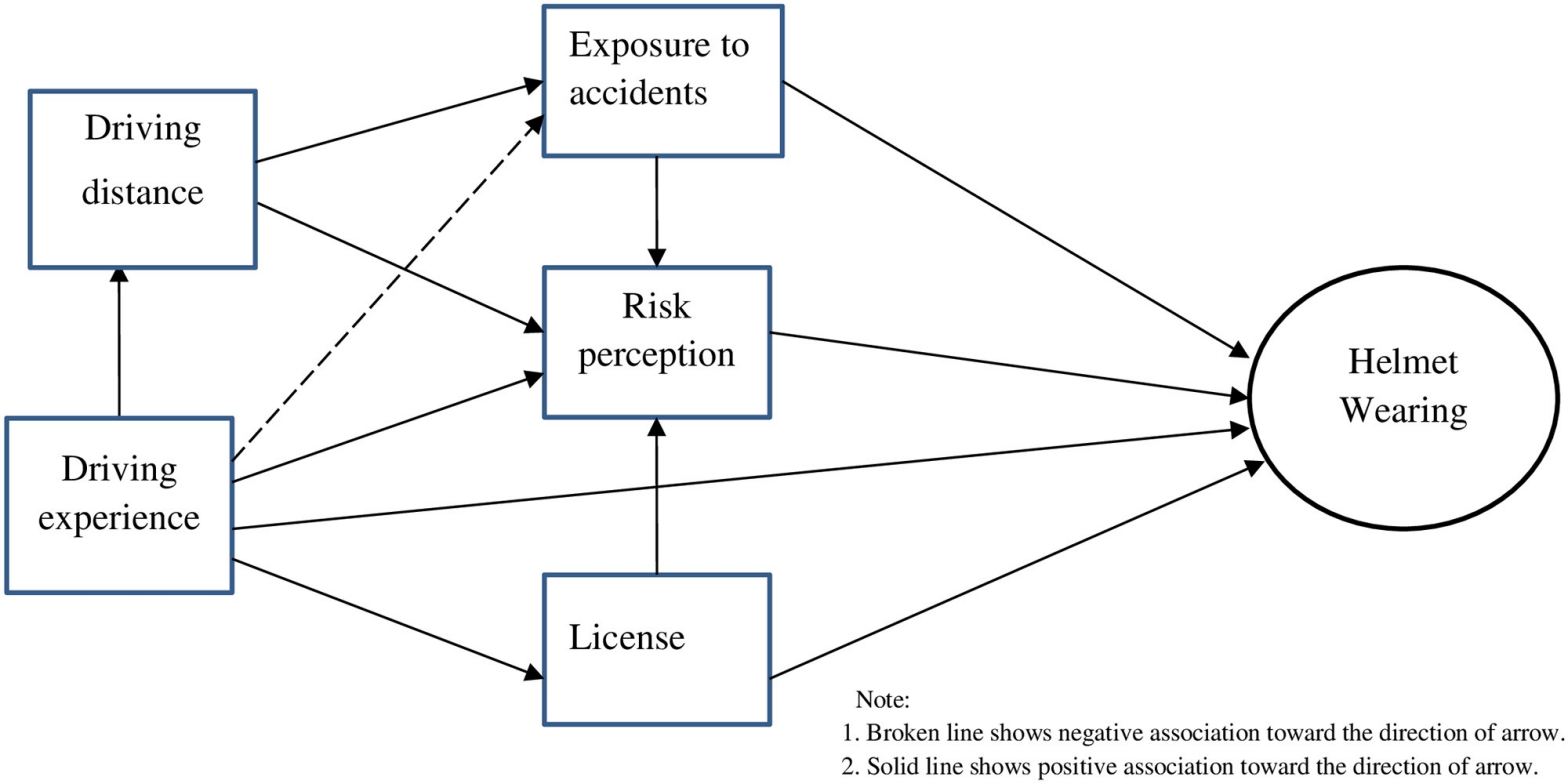
Hypothesized relationships among factors.

Fig 3 shows the hypothesized relationships among the variables. We hypothesized that long-distance driving had a positive association with perceived risk and actual exposure to accidents. As people drive long distances, their actual exposure to accidents and the perceived risk of exposure to accidents increase. Driving experience had a positive association with perceived risk of exposure to accidents and having motorcycle license. However, driving experience may have a negative relationship with exposure to accidents. We hypothesized that driving distance had an indirect association with wearing helmets. Exposure to accidents, risk perception and having a license had direct association with wearing helmets. Driving experience had both direct and indirect associations with helmet wearing (Fig 3).

## Discussion

Road traffic accidents have been increasing in both developed and developing countries. Motorcycles are among the main contributors to road traffic accidents especially in low- and middle-income countries where motorcycles are the primary mode of transportation. Wearing personal protective devices especially helmets is the most cost-effective and has the potential to substantially reduce road traffic injuries, disabilities, deaths and associated personal and societal costs.

### Prevalence of helmet wearing behavior

The findings of our study showed that only 12.4% of the study participants wore helmets. This finding was far lower than that reported in studies conducted in high-income countries such as the united states (68.4%) [38] and Australia (89%) [39]. This might be due to the differences in the study population. The current study was conducted on all types of riders. However, a study from the United States was conducted among recent graduates of a motorcycle training course, and a study from Australia was conducted among cyclists who faced crashes in transport-related areas. Attending training increases knowledge, perceived benefit of the desired behavior, positive attitude and finally helps individuals to engage in the intended behavior/helmet wearing. A history of accidents might also enable people to engage in the recommended behavior. The discrepancy might also be due to the differences in legislation on helmet wearing. The finding of our study was also lower than those of studies conducted in upper-middle-income countries such as Mexico (73.8%) [40], India (64.7%) [41], Thailand (55.8%) [42], China (43.2%) [43] and Argentina (40%) [44]. This discrepancy might be due to differences in helmet laws. Helmet wearing is mandatory for both motorcycle drivers and pillion riders in these countries unlike in Ethiopia and law enforcement strictly has been on penalties for motorcyclists who do not obey helmet use and even seize their vehicles. The existence of such legislation enforces riders to wear personal protective equipment and helps to reduce road traffic accidents. The discrepancy might also be due to the strong operationalization of the behavior (helmet wearing) in terms of frequency, duration and purpose in our study. This means, only motorcycle riders who always wear helmets in the last three months before the study period for the sake of reducing injuries and other consequences of road traffic accidents were considered as “wear helmet” in our study. Indeed, this difference might also be due to the sample size difference. The finding of the current study was comparable with studies conducted Iran where (10%) [45] and (10.7%) [46] of motorcyclists wore a standard helmet while riding. However, the finding of our study was lower than those of studies conducted in low-middle income countries such as Cameroon (65%) [47], Pakistan (56%) [48], Myanmar (51.5%) [49], India (44.5%) [50], Vietnam (23%) [51], Ghana (47%) [52], Tanzania (42.3%) [53], Kenya (28%) [54], and a study from a low-income country (Uganda) where 30.8% of drivers wore helmets [37]. This might be due to the relatively strong legislation of helmet wearing in these countries compared to Ethiopia. In Ethiopia, wearing helmet is not mandatory and people wear as per their interests. The discrepancy might also be due to the strong operationalization of the behavior (helmet wearing) in terms of frequency, duration and purpose in our study. However, the finding of our study was higher than those of a study from Nigeria, where only 2.7% of participants wore helmets regularly [55] and a study from Malawi which showed that of the 1900 cyclists observed, no cyclist was identified as wearing helmet [56]. This might be due to the differences in the study population. A study conducted in Malawi was conducted among bicycle riders while our study was conducted among motorcycle riders. This discrepancy might also be due to gaps of study period between the previous studies and current study.

### Factors associated with helmet wearing behavior

The current study identified the determinants of helmet wearing among motorcycle riders. In this study, having a license most predicted helmet wearing behavior. The likelihood of wearing helmets was nearly 3.5 times higher among motorcycle drivers who had licenses compared to their counterparts. This finding was similar to studies conducted in the United Kingdom [57], Southern Iran [58] and Ghana [52, 59]. This might be due to the fact that license owners took training on traffic rules and ways of reducing road traffic accidents and the benefits of using personal protective equipment such as helmets. Hence training helps to increase knowledge about helmet wearing, attitude and intention towards helmet wearing, and finally the behavior that is wearing helmets. Thus, relevant key authorities like traffic police should have to allow only riders with a motorcycle license to ride in order to encourage others to apply for a license. Indeed, conducting public education like awareness creation campaigns, promoting motorcycle riders training programs, routine sensitization on safe riding and giving attention to road safety measures to improve helmet wearing among motorcyclists need to be focused especially in low income countries like Ethiopia where motorcycle accidents are highly prevalent. Studies from Thailand [42] and Uganda [60] pointed out the association between training and road safety compliance, helmet use.

In the current study, risk perception was the second most significant predictor of helmet wearing behavior. Motorcycle drivers who had high perceived susceptibility to accidents were 3.1 times more likely to wear helmets compared to those who had low perceived susceptibility to accidents. This was due to the fact that acceptable risk perception can help individuals to take protective measures. However, a highly increased risk perception may cause fear, tension, and depression which leads to ignorance of the desired behavior. A study from the United Kingdom showed an association between risk perceptions and motorcycle drivers’ non-personal protective equipment wearing [57]. However, the study from United Kingdom assessed general personal protective equipment and not specifically focused on helmet wearing. Thus, concerned bodies should prepare and distribute advocacy messages about the risks and consequences of motorcycle accidents.

The findings of the current study also indicates an association between helmet wearing and driving experience. Motorcycle drivers with driving experience of ≥10 years had higher odds of wearing helmets compared to those with < 5 years of driving experience. This finding was supported by studies conducted in Iran [58] and Batu Pahat, Johor [61]. This may be due to the fact that as experience increases, the chance of obtaining right information also increases, which may increase individuals’ perceived risk of accidents and motivate them to engage in wearing helmets. Similar to a study conducted in Vietnam [51], our study also showed that motorcycle drivers who had a history of exposure to motorcycle accidents were more likely to wear helmets compared to those who had no history of motorcycle accidents. This was probably due to the previous exposure to an accident can increase individuals’ perception of the risks and seriousness of the accidents. In addition, exposure to accidents may also increase the perceived benefits of using personal protective devices, which may encourage riders to wear helmets. Thus, there is a need for mandatory universal helmet legislation and periodic police checks especially in countries where motorcycle accidents are highly prevalent.

In this study, the riding distance also determined the helmet wearing behavior of motorcycle riders. Motorcycle riders who commonly drove a distance of >10Km were more likely to wear helmets compared to those who drove a distance of ≤10 Km. This finding was in agreement with studies conducted in Vietnam [51] and Batu Pahat, Johor [61], which showed higher compliance to safety helmet usage among motorcyclists who travel a longer distance. The higher usage of safety helmets for longer distance trips might be due to a higher perceived risk of accidents. Indeed, driving longer distances may also increase the chance of exposure to road traffic accidents which may in turn increase drivers’ risk perception, motivation and finally utilization of helmets.

### Strengths and limitations of the study

This study has several strengths. One of the strengths of this study is that the authors defined the behavior (helmet wearing) in terms of frequency, duration, and purpose. This means motorcycle riders were considered wearing helmets if they were always wearing helmets for the sake of reducing motorcycle injuries while they drive in the last three months before the study period. To the best of the authors’ knowledge, this was the first study to elicit responses related to helmet wearing in Ethiopia. Thus, this study contributes to the limited literature. This study has also several limitations. Hence, the findings of the study should be interpreted in light of the following limitations. First, As sampling frames of motorcycle drivers were prepared based on the registered motorcycle, those who drive unregistered motorcycles were not included in this study. Thus, it was difficult to generalize the findings of this study to all motorcycle drivers of Sawula and Bulky towns. Second, Due to the nature of the study, there might be interviewee bias as some respondents were interviewed at their workplace and busy with their work. However, to reduce this, participants were well informed about the purpose of the study and the data collectors scheduled the time of interview after discussing the study participants. Third, Recall bias might occur for some variables. Fourth, there might be social desirability bias for interviews conducted at police stations. However, to reduce this, training was given for data collectors and supervisors to keep their distance from crowded areas and influential people like traffic police during interviews with the study participants.

## Conclusion

This study found that helmet wearing was very low. Having a license, driving strips, exposure to accidents, driving experience and accident risk perception were determinants of helmet wearing behavior. These determinants imply that helmet wearing relies mainly on motorcycle drivers’ knowledge and perceptions about accidents and helmet wearing. Thus, we recommend behavioral change communication interventions that focus on increasing knowledge about helmet wearing and the perceived risk of motorcycle accidents using different strategies such as awareness creation campaigns, media, and mandatory helmet wearing laws and applications for license. We also advise researchers to measure helmet wearing status of pillions or passengers.

## Supporting information

S1 File (DOCX)

## Abbreviations

AOR: Adjusted odds ratio
CI: Confidence interval
COR: Crude odds ratio
RTA: Road traffic accidents
