# Supplementary material for: Helmet wearing behavior where people often ride motorcycle in Ethiopia: A cross-sectional study
Source: PLoS One. 2022 Jan 27;17(1):e0262683. doi: 10.1371/journal.pone.0262683 (PMC8794078; doi:10.1371/journal.pone.0262683)
Supplement: S1 File — (DOCX) [file pone.0262683.s001.docx]

**Part I: Socio-demographic and economic factors**

| **S. No** | **Variable** | **Response options** |
| --- | --- | --- |
| 100 | Where is your working organization? | 1. Private 2. Government 3. NGO |
| 101 | Sex of the respondent | 1. Male 2. Female |
| 102 | Age of respondent | ______Years |
| 103 | What is your marital status? | 1. Single  2. Married  3. Divorced  4. Separated  5. Widowed |
| 104 | What is your level of education? | 1. Unable to read and write  2. Able to read and write  3. 1-8 Grade  4. 9-12 grade  5. Diploma/technical/vocational  6. Degree and above |
| 105 | What is your monthly income? | ___________(ETB) |
| 106 | What is your occupation? | 1. Commercial motorcycle driver  2. Farmer  3. Merchant  4.Government employee  5. Other (specify)____________ |

**Part II: Driving related factor**

| **S. No** | **Question** | **Response options** | **Remark** |
| --- | --- | --- | --- |
| 201 | Do you ever have a motorcycle traffic accident? | 1. Yes 2. No |  |
| 202 | For how long is your driving experience as a motorcycle driver? | _____years |  |
| 203 | Do you have a motorcycle driving license? | 1. Yes 2. No |  |
| 204 | Do you have your own motorcycle? | 1. Yes 2. No |  |
| 205 | Where do you drive most? | 1. Inside the main road 2. Outside the main road |  |
| 206 | On average how many kilometers do you drive in a day in a single trip mostly? | ________Km |  |
| 207 | How often do you drive a motorcycle? | 1. Daily 2. Sometimes 3. Rarely |  |

**Part III: Substance use**

| **S. No** | **Variable** | **Response options** | **Remark** |
| --- | --- | --- | --- |
| 301 | Do you drink alcoholic beverages? | 1. Yes 2. No | If 2, skip to 304 |
| 302 | Have you drunk any alcoholic beverage in the past 3 months? | 1. Yes 2. No | If 2, skip to 304 |
| 303 | Have you driven within 4 hours after you drunk alcoholic beverages in the past 3 months? | 1. Yes 2. No |  |
| 304 | Do you chew chat? | 1. Yes 2. No | If 2, skip to 401 |
| 305 | Have you chewed chat in the past three months? | 1. Yes 2. No | If 2, skip to 401 |
| 306 | Have you driven after chewing chat in the past three months? | 1. Yes 2. No |  |

**Part IV: Perceptions**

The following items are prepared to measure perceived susceptibility and perceived severity of motorcycle accidents. The response options are: Strongly Disagree (SDA)=1, Disagree (DA)=2, Neutral(N)=3, Agree (A)=4 and Strongly Agree (SA)=5.

| **S. No** | **Items** | **Response options** | | | | |
| --- | --- | --- | --- | --- | --- | --- |
|  |  | **SD** | **D** | **N** | **A** | **SA** |
|  | **Perceived susceptibility** |  |  |  |  |  |
| 401 | I feel I will get motorcycle accidents during my life. |  |  |  |  |  |
| 402 | Small exposures to motorcycle driving won’t lead me to accidents. |  |  |  |  |  |
| 403 | I worry a lot about becoming injured in driving motorcycle. |  |  |  |  |  |
| 404 | As a motorcycle rider, the probability of being injured in motorcycle accident is very high. |  |  |  |  |  |
|  | **Perceived severity** |  |  |  |  |  |
| 405 | If I got an accident without wearing helmet, I would feel that I would have a serious injury. |  |  |  |  |  |
| 405 | If I ride without wearing helmet, I would feel that I will get death due to serious injuries. |  |  |  |  |  |
| 407 | Being injured in motorcycle accidents because of not wearing helmet could lead to long-standing problems. |  |  |  |  |  |
| 408 | When I think about motorcycle accidents that occur without wearing helmet, my heart beats faster. |  |  |  |  |  |

**Part V: Social Pressure**

The following items are prepared to measure social pressures related to wearing helmet. The response options are: Strongly Disagree (SDA)=1, Disagree (DA)=2, Neutral(N)=3, Agree (A)=4 and Strongly Agree (SA)=5.

| **S/No** | **Items** | **Response options** | | | | |
| --- | --- | --- | --- | --- | --- | --- |
|  |  | **SD** | **D** | **N** | **A** | **SA** |
| 501 | I know that I will feel bad if I don’t wear helmet because my parents or somebody that cares about me want to wear it. |  |  |  |  |  |
| 502 | I have friends that encourage me or sometimes remind to wear helmet when I ride. |  |  |  |  |  |
| 503 | I think I should keep myself safe for the people who care about me by wearing helmet when I ride. |  |  |  |  |  |
| 504 | Some of my family thinks I should wear helmet when I drive a motorcycle. |  |  |  |  |  |
| 505 | I am sensitive to the trends established by my friends and I usually try to follow them so I won’t be seen as being too different. |  |  |  |  |  |

**Part-VI: Knowledge about helmet wearing**

| **S. No** | **Questions** | **Response options** |
| --- | --- | --- |
| 601 | Have you heard about helmet which is prepared for motorcycle drivers to wear during driving? | 1. Yes 2. No |
| 602 | If yes, from where do you heard? | 1. Mass media  2. Family or friends  3. Training  4. Other (specify)________ |
| 603 | Wearing a full-faced helmet is protective for head injury | 1. Yes 2. No |
| 604 | Wearing a full-faced helmet is protective for facial injury | 1. Yes 2. No |

**Part-VII: Helmet wearing behavior**

| **S. No** | **Question** | **Response options** |
| --- | --- | --- |
| 701 | Do you have helmet prepared for motorcycle drivers to use during driving? | 1.Yes  2. No |
| 702 | Have you worn helmet prepared for motorcycle drivers in the past three months during driving for the sake of reducing injuries due to motorcycle accidents? | 1.Yes  2. No |
| 703 | If yes, how often do you wore? | 1. Always  2. Sometimes  3. Rarely |
